# Solid Pseudopapillary Neoplasms of the Pancreas: A Report of Two Cases

**DOI:** 10.1155/2014/356379

**Published:** 2014-06-01

**Authors:** Dilip Dan, Rakesh Rambally, Shamir O. Cawich, Ravi Maharaj, Vijay Naraynsingh

**Affiliations:** Department of Clinical Surgical Sciences, University of the West Indies, St. Augustine Campus, St. Augustine, Trinidad and Tobago

## Abstract

Solid pseudopapillary neoplasms of the pancreas are uncommon, accounting for only 1-2% of all pancreatic neoplasms. These tumors are being detected at an increased rate, probably due to the increased awareness and the liberal use of imaging. We report two cases of patients with solid pseudopapillary pancreatic tumors and review the existing literature.

## 1. Introduction

Solid pseudopapillary neoplasms (SPNs) are uncommon tumors, accounting for only 1-2% of pancreatic neoplasms [1, 2]. Although pancreatic SPNs are uncommon, clinicians should consider the diagnosis in young women with typical lesions because these patients have good outcomes with appropriate treatment.

## 2. Report of Cases

### 2.1. Case 1

**Figure 1:**
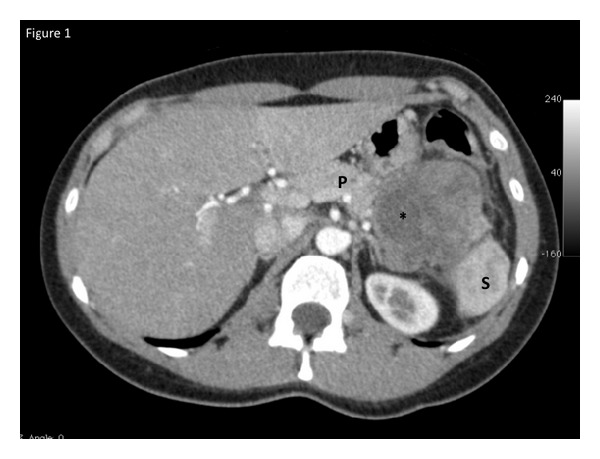
Contrast enhanced CT scan of the abdomen revealing the solid pseudopapillary tumor in the pancreatic tail (P). The spleen (S) is visible distally and an area of cystic degeneration is noted centrally marked by an asterix.

A 35-year-old woman of East Indian descent presented to a hospital with a long-standing complaint of vague epigastric discomfort for 18 months. She noted that the upper abdomen became “full” over this time but there were no other symptoms present. After an abdominal ultrasound suggested the presence of a pancreatic tumor, a multiphase contrast-enhanced computed topography (CT) scan was ordered (Figure 1).

The CT scan images revealed a well-circumscribed lesion in the pancreatic tail that measured approximately 6 cm in diameter. There were peripheral enhancement and a central area of cystic degeneration present. Minimal calcifications were noted. Serum assays of carcinoembryonic antigen and CA 19-9 were within normal levels.

Based on characteristic findings on cross-sectional imaging in this young female, a diagnosis of pancreatic SPN was entertained and the patient was taken to the operating room for a distal pancreatectomy. This was completed uneventfully using the laparoscopic approach, with tumor extraction through an upper midline incision. A 19 Fr drain was placed at the pancreatic bed. The patient recovered uneventfully. The drain was removed on the fourth postoperative day and the patient was discharged home shortly after.

**Figure 2:**
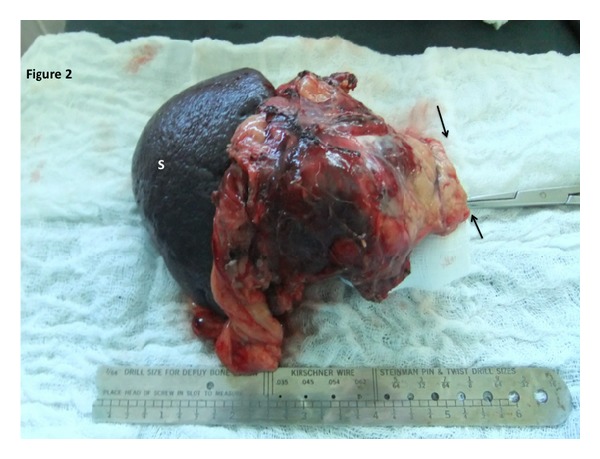
The specimen has been resected en bloc with the spleen (S) through a laparoscopic approach. Black arrows point to the macroscopically clear resection margin at the pancreatic tail.

On gross pathologic examination, an encapsulated tumor 60 mm in maximal diameter was seen in the tail of the pancreas (Figure 2). There was a distance of 1 cm between the tumor and the pancreatic resection margins. The tumor was composed of small polygonal cells with small centrally placed nuclei. Histiocytes with large inclusion vacuoles within their cytoplasm were seen occasionally. Centrally, there were multiple areas of tumor necrosis with cystic degeneration. There was an area of haemorrhagic necrosis that obscured the capsule at the distal margin. There were also areas of vascular invasion noted on high power examination. The cells stained positively for antitrypsin, vimentin, and neuron specific enolase on immunohistochemistry. All other stains were negative.

A diagnosis of a pancreatic SPN was made and this patient underwent adjuvant systemic treatment with intravenous gemcitabine. After four years of surveillance, there has been no evidence of local or systemic disease recurrence in this patient.

### 2.2. Case 2

**Figure 3:**
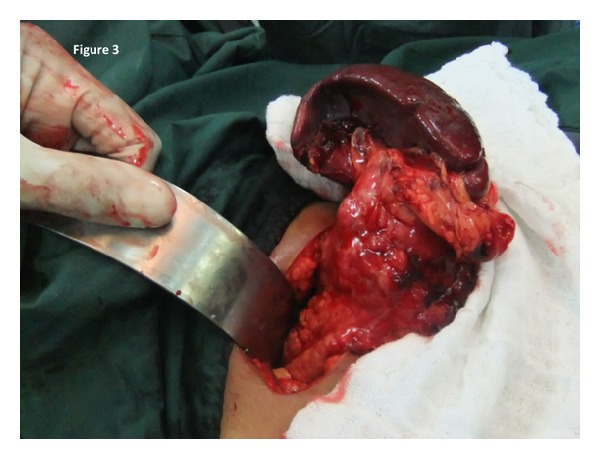
Open pancreatosplenectomy being performed through a subcostal incision in the left upper quadrant.

A 26-year-old woman had been experiencing vague dyspeptic symptoms, nausea and vomiting, for three months. She was sent for an abdominal ultrasound to confirm a clinical diagnosis of symptomatic cholelithiasis. Ultrasound revealed a discrete pancreatic tail mass, approximately 2.5 cm in diameter. There was a characteristic cystic degeneration with associated calcifications centrally within the lesion. Tumor markers were within normal limits. A distal pancreatosplenectomy was again performed, this time using the open approach. The splenic artery and vein were both suture ligated and pancreatic tail was transected. A 19 Fr. drain was left at the resection margin. The spleen and pancreatic tail were excised en bloc through the left upper quadrant subcostal incision (Figure 3). Postoperative recovery was uneventful, with hospital discharge on day 4 and drain removal on day 8 after operation.

**Figure 4:**
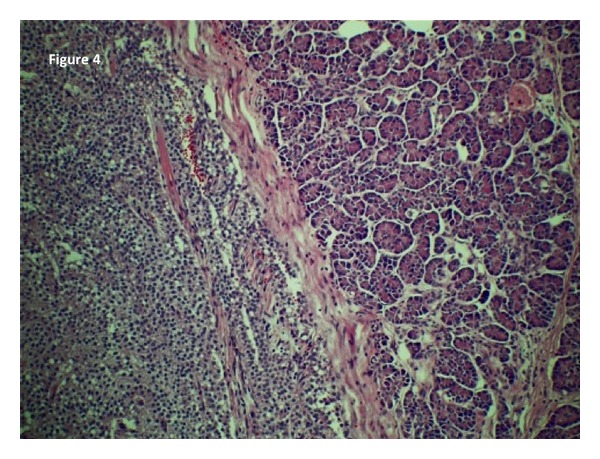
Microscopic view of the solid pseudopapillary tumor within the pancreas. The fibrous capsule can be visualized centrally separating normal acini. Numerous small ovoid cells with prominent nuclei are seen within the capsule. No mitoses are seen and there is no evidence of vascular or capsular invasion.

On gross pathologic examination of the excised specimen, a 21 mm encapsulated tumor was noted in the pancreatic tail 2.2 cm from the resection margin. The cut surface was tan-brown in color and there were focal areas of central necrosis and haemorrhage. Associated with the necrosis, there were areas of cystic degeneration. On microscopic examination, the tumor was composed of regular ovoid cells with small central nuclei and eosinophilic abundant cytoplasm (Figure 4). There were no mitoses present and no capsular, lymphatic, or vascular invasion was seen. Immunohistochemistry staining was positive for neuron specific enolase and synaptophysin but negative for CA 19-9, carcinoembryonic antigen, alpha-1-antitrypsin, vimentin, and chomogranin.

A diagnosis of pancreatic SPN was made. The resection margins were microscopically clear and no further treatment was offered to this patient. Two years after resection, there is no evidence of local recurrence and the patient remains symptom free.

## 3. Discussion

Solid pseudopapillary neoplasms of the pancreas are uncommon lesions, accounting for 1-2% of pancreatic neoplasms [1, 2]. There has been an apparent increase in the incidence of pancreatic SPNs over the past two decades, but this is likely due to the increased use of advanced imaging modalities rather than a genuine increase.

Some credit Lichtenstein [3] with the first description of SPN when he reported a large pancreatic tail mass in a young woman with peritoneal carcinomatosis. However, the first unequivocal account was a series of three cases with detailed pathologic descriptions by Frantz in 1959 [4]. Hamoudi et al. [5] were the first to characterize the pathognomonic electron microscopic features of pancreatic SPNs. For this reason, some refer to these lesions as Frantz tumors or Hamoudi-Frantz tumors.

There are several case reports and small series of pancreatic SPN in the world literature. The largest series comprised 718 cases and was published by T. Papavramidis and S. Papavramidis in 2005 [1]. They noted that the majority of the reports originated from Europe, Japan, and North America. To the best of our knowledge, this entity has only been reported once from the Caribbean region [6].

There is a marked predilection for pancreatic SPNs to occur in young women and adolescent girls. In Papavramidis' series, the diagnosis was made at a mean age of 22 years (range 2–85) and there was a tenfold female preponderance [1]. Machado et al. [7] also noted that the presentations differed by gender, with the diagnosis being made a decade earlier in females than their male counterparts (25 years versus 37 years). Both cases occurred in east Indian women while our population is comprised of equal proportions of persons of Afro- and Indio-Trinidadian descent [8]. This was interesting, but there is no recognized racial predilection for SPNs.

The exact cell of origin is still disputed [9]. The strong female predilection [1, 2] and the reported expression of progesterone receptors in some cases [10] may suggest an association between female sex hormones and tumorigenesis, but a causal relationship has not been definitively proven.

Pancreatic SPNs are usually indolent tumors. As such, they tend to produce vague nonspecific symptoms or may be detected incidentally on imaging. Less than 10% of patients are symptomatic on presentation [11], with the commonest symptom being vague abdominal pain [12]. Although the majority of cases are asymptomatic, both patients in our series presented with vague upper abdominal pain.

As these lesions enlarge, they may then cause symptoms from mass effect, such as vomiting and early satiety due to gastric outlet obstruction. Jaundice is not a common feature—although there is an equal incidence of SPNs in the pancreatic head and tail. In Papavramidis' series [1], only 1% of patients with pancreatic head tumors were jaundiced.

The lesions may enlarge significantly to become noticeable on inspection or detected on palpation. Interestingly, when Seung et al. [13] compared the clinical features between adults and children, they noted that children had a larger mean tumor diameter at presentation (8 cm versus 6 cm) and were more likely to have a palpable mass.

There are no pathognomonic features on blood investigations and tumor markers are usually unremarkable. The diagnosis is usually made on cross-sectional imaging when pathognomonic features are present [14]: encapsulated, well-defined mass with central areas of calcification, necrosis, haemorrhage, and/or cystic degeneration. In both the arterial and venous phases, there is usually peripheral enhancement with similar hounsfield unit density as the nearby pancreatic parenchyma [14]. This differs from adenocarcinomas that usually are hypoattenuated on venous phase CT and from pancreatic neuroendocrine tumors (pNET) that enhance on the arterial phase CT [15]. The diagnosis can usually be made on multiphase contrast enhanced CT with an estimated 60% overall accuracy [15], but some authorities also advocate magnetic resonance imaging because it may be more able to delineate tissue characteristics such as haemorrhage and necrosis [16]. We found MRI unnecessary in our experience because a contrast enhanced CT scan was sufficient to comfortably make the diagnosis in both of our cases.

After the diagnosis is made on cross-sectional imaging, young patients with good performance status should be managed operatively. Preoperative histologic confirmation is not necessary and is usually reserved for patients who have high operative risk or who require complex resections (borderline-resectable tumor, vascular resection required, and/or nodal disease outside of the resection margins). In these cases, image guided fine needle aspiration cytology may make the diagnosis preoperatively with 62–70% overall accuracy [11, 12, 17]. The lesion is comprised of small ovoid or polygonal cells with small central nuclei and abundant cytoplasm [17, 18]. Histologic markers of poor prognosis include a high mitotic rate, spindling of tumor cells, anaplastic giant cells, capsular invasion, and lympho-vascular involvement [17–20].

Image guided core biopsy can also be considered in challenging cases since the yield may be sufficient to allow immunohistochemical staining. Over 90% of these tumors stain positively for vimentin, neuron specific enolase, alpha-1-antitrypsin, alpha-1-antichymotrypsin, and/or progesterone receptors [18]. Other immunohistochemical features include nuclear localization of beta-catenin [19, 20] and loss of E-Cadherin from the cytoplasmic membrane [21]. But these tumors do not stain for chromogranin, CK19, or acinar cell markers such as trypsin [22].

Patients with resectable lesions who are candidates for operation should be treated by en bloc resection with clear margins, since this provides the best chance for a cure. These tumors are clinically indolent [11] and there are many reports of long-term survival after complete resection in excess of five years [9, 11, 12, 23, 24].

Even in the presence of poor prognosticators (lympho-vascular invasion, capsular invasion, local extension, nodal disease, and liver metastases) that traditionally predict malignant behavior [12, 23], there are still relatively good outcomes compared to adenocarcinomas. Long-term survival has been documented despite the presence of these prognosticators [23–26]. Therefore, many surgeons advocate aggressive resections, even in the face of extrapancreatic disease [11, 12, 23–26].

The extent of resection is still debated. In a retrospective series of 34 patients, Li et al. [27] compared “standard” and “minimized” pancreatic resections for SPTs. Both groups had similar morbidity rates and long-term survival, but patients subjected to “standard resections” had longer operating times (225 versus 124 minutes; *P* = 0.004), transfusion rates (53% versus 13%; *P* = 0.03), and hospitalization (21 versus 16 days; *P* = 0.034). Based on these preliminary data, Li et al. [27] advocated “minimized resections, such as enucleation.” However, their mean follow-up duration was only 29 months, which is insufficient to demonstrate a difference in long-term survival. Most scientific data consider five-year survival rates as an outcome marker [28]. Additionally, they advocated enucleation on the basis of these data, but their “minimized resection group” included 9 enucleations and 6 segmental resections. There are no large prospective randomized trials comparing the degree of resection, and it is probably not feasible considering the rarity of pancreatic SPTs. However, there are existing data demonstrating that the malignant potential of SPTs cannot be reliably predicted on preoperative data such as gender [28, 29], age [16, 28–30], tumor size [28], CT findings [26] tumor markers [16, 25, 27], biopsy findings [9, 16], or immunohistochemical patterns [29]. Therefore, most authorities advocate formal R0 resections aiming for clear microscopic margins [2, 9, 24–30].

There is also an ongoing debate about the ideal operative approach to SPTs. When Zhang et al. [31] retrospectively compared open and laparoscopic operations in 28 patients who required distal pancreatectomy for SPTs, they reported that both approaches had similar operation times, postoperative morbidity, mortality, reoperation rates, nodal harvest, margin clearance, and 3-year survival. But the laparoscopic approach had definite short-term advantages, with significantly lower blood loss (149 mL versus 580 mL; *P* = 0.002), transfusion requirements (7% versus 46%; *P* = 0.029), resumption of oral intake (2.3 versus 4.9 days, *P* < 0.001), and hospitalization (8.1 versus 12.8 days, *P* = 0.029). Several other studies have also proved that the laparoscopic approach is safe and oncologically adequate [32, 33], although it should be reserved for trained laparoscopic surgeons due to the technical complexity of these operations [32]. For this reason, only one case was approached laparoscopically in our series.

There is no good comparative data evaluating the role of systemic chemotherapy or the optimized agents. Only small individual case reports are available [26, 29, 34–37]. Therefore, the decision to administer systemic therapy is usually an individualized one. Although some advocate systemic therapy when poor prognosticators [26, 34–37] or metastatic disease [36–38] is present, there is no existing data to prove long-term survival benefit in these patients [39]. There have also been individual case reports of gemcitabine being used to achieve downsizing for initially unresectable disease [40, 41]. In our first case, we administered gemcitabine as adjuvant systemic therapy because poor prognosticators were noted (capsular and vascular invasion), but we concede that this decision could be challenged easily since there is no existing firm data to support this.

## 4. Conclusion

Pancreatic SPNs are rare neoplasms with malignant potential found primarily in young women. Formal surgical resection may be performed safely and is associated with long-term survival. Because long-term survival can be achieved, patients with SPN should be treated aggressively with complete resection, even if this requires metastasectomy.
